# Development, Pathogenesis, and Regeneration of the Intervertebral Disc: Current and Future Insights Spanning Traditional to Omics Methods

**DOI:** 10.3389/fcell.2022.841831

**Published:** 2022-03-11

**Authors:** Tara T. Hickman, Sudiksha Rathan-Kumar, Sun H. Peck

**Affiliations:** ^1^ Department of Medicine, Vanderbilt University Medical Center, Nashville, TN, United States; ^2^ Division of Clinical Pharmacology, Vanderbilt University Medical Center, Nashville, TN, United States; ^3^ Vanderbilt Center for Bone Biology, Vanderbilt University Medical Center, Nashville, TN, United States

**Keywords:** intervertebral disc, animal models, regeneration, development, omics, degeneration, therapies

## Abstract

The intervertebral disc (IVD) is the fibrocartilaginous joint located between each vertebral body that confers flexibility and weight bearing capabilities to the spine. The IVD plays an important role in absorbing shock and stress applied to the spine, which helps to protect not only the vertebral bones, but also the brain and the rest of the central nervous system. Degeneration of the IVD is correlated with back pain, which can be debilitating and severely affects quality of life. Indeed, back pain results in substantial socioeconomic losses and healthcare costs globally each year, with about 85% of the world population experiencing back pain at some point in their lifetimes. Currently, therapeutic strategies for treating IVD degeneration are limited, and as such, there is great interest in advancing treatments for back pain. Ideally, treatments for back pain would restore native structure and thereby function to the degenerated IVD. However, the complex developmental origin and tissue composition of the IVD along with the avascular nature of the mature disc makes regeneration of the IVD a uniquely challenging task. Investigators across the field of IVD research have been working to elucidate the mechanisms behind the formation of this multifaceted structure, which may identify new therapeutic targets and inform development of novel regenerative strategies. This review summarizes current knowledge base on IVD development, degeneration, and regenerative strategies taken from traditional genetic approaches and omics studies and discusses the future landscape of investigations in IVD research and advancement of clinical therapies.

## Introduction

The intervertebral disc (IVD) is a fibrocartilaginous structure that connects the vertebral bodies in the spine. The IVD is composed of three substructures: 1) the proteoglycan-rich central region referred to as the nucleus pulposus (NP), 2) the fibrocartilaginous peripheral region referred to as the annulus fibrosus (AF), and 3) the superior and inferior cartilaginous endplates (CEP) (Humzah and Soames, 1988; Cassidy et al., 1989). The main role of the IVD is to provide structural support to the spinal column by absorbing and transferring the compressive forces that are applied to the spine and to confer mobility to the spine.

The IVD is particularly prone to impaired repair leading to permanent damage. The IVD, which is the largest avascular organ in the body, has low cell density, low nutrient flow, and a low oxygen environment, creating a particularly harsh microenvironment for the resident cells (Diamant et al., 1968; Ishihara and Urban, 1999). Thus, these cells tend to be much less metabolically active, even in healthy states (Bibby et al., 2005). IVD degeneration (IDD) and associated back pain is a major socioeconomic burden, affecting 85% of the population at some point in their lifetime and costing over $100 billion annually in the United States (Katz, 2006; Hoy et al., 2014; Buchbinder et al., 2018). While IDD has been implicated as a major cause of back pain, the direct mechanistic links to pathology have not been fully elucidated. Current treatments for back pain include physical therapy, anti-inflammatory medications, and in severe cases, artificial disc replacement or spinal fusion (Mirza and Deyo, 2007). None of these treatment approaches recapitulate native structure and function of the disc, and the primary focus is on alleviating or managing pain. Thus, there is a need for development of therapies that can restore disc structure and functional mechanics while alleviating the painful symptoms associated with IDD and back pain. As such, there has been much interest in developing regenerative therapies to apply to the IVD.

Regenerative strategies that have garnered much interest in recent years are focused on elucidating the native developmental processes of the IVD to apply early developmental signals for *in vivo* tissue regeneration by combining tissue engineering using biomimetic scaffolds, targeted drug delivery for endogenous cell activation and differentiation, and exogenous cell implantation (Pirvu et al., 2014; Pirvu et al., 2015; Richardson et al., 2016; Bowles and Setton, 2017; Baumgartner et al., 2021; Du et al., 2021). Improved understanding of intervertebral disc formation may enable reproduction of important developmental signals in the degenerated or injured discs as a regenerative strategy. Elucidating a fuller profile of the specific molecular signals that direct tissue formation and applying these signals to therapeutic cell types like mesenchymal stem cells (MSCs) or induced pluripotent stem cells (iPSCs) could potentially drive these cells towards AF or NP-like phenotypes that can regenerate the damaged tissue. While many laboratory studies have pointed to potential successful application of pluripotent and multipotent therapeutic cell types in regenerative medicine, many important considerations remain under investigation. Fully pluripotent stem cells like embryonic stem cells (ESCs) are theoretically the ideal cell type in terms of regenerative potential, but ethical concerns, limited cell numbers and sources for harvest, and immunologic reactions after implantation hinder application of these cells. On the other hand, MSCs are plentiful and do not carry the same ethical burdens as they can be harvested from multiple tissues even from adult donors and seem to induce minimal or no immunologic response. However, limited proliferative capability and cell senescence remain hurdles for widespread application of MSCs. Importantly, the IVD is a particularly harsh environment for implanted cell survival, differentiation, and highly synthetic processes like tissue formation, and as such, continued studies delving into the multi-faceted aspects of the IVD to inform effective therapeutic development have remained a hotly pursued area of investigation.

To this end, animal models, particularly genetic mouse models, have provided critical insights into genetic and molecular profiles of IVD development. In addition to murine models, larger animals such as rabbit, dog, sheep, and bovine have played an important role as their IVDs are more similar in size and mechanical load to human IVDs, particularly in studies involving biomechanical changes over time and the application of pre-clinical therapies (Alini et al., 2008; Sakai and Andersson, 2015). While challenges in translating findings from animal models to human patients due to differences in important factors such as IVD size, cell turnover, and biomechanical load need to be acknowledged and carefully considered, the usefulness of these experimental models cannot be ignored. Furthermore, the advent of omics technologies like transcriptomics, proteomics, and metabolomics have allowed researchers to make significant progress in understanding global profiles and longitudinal changes in the IVD, both during development and in degeneration. Although human samples are much more difficult to collect, especially in large cohorts, these technical advances have allowed researchers to gain insight into gene and protein expression profiles of the human IVD at different developmental stages as discussed below. With these omics technologies becoming widely available to most research labs, both in terms of cost and accessibility, in-depth analyses of IVD animal models have become commonplace in recent years. This allows for wide-reaching comparative studies between different types of animal models as well as comparisons with findings from human patient samples, all efforts that have been pointing the IVD research community toward better consensus on important topics such as the molecular basis of IVD development, maintenance, and degeneration, and molecular markers that define various cells and components within the IVD. Collectively, the combined growing breadth and depth of available data in all aspects of IVD research from both human patient samples and animal models will inform novel diagnostics and therapeutics that will ultimately address the major clinical need of developing long-term IDD treatments.

This review summarizes the currently known aspects of IVD development, degeneration, and therapeutics taken from traditional and omics studies as well as the future landscape of clinical treatment and regenerative strategies in IVD research.

### Intervertebral Disc Composition

**FIGURE 1:**
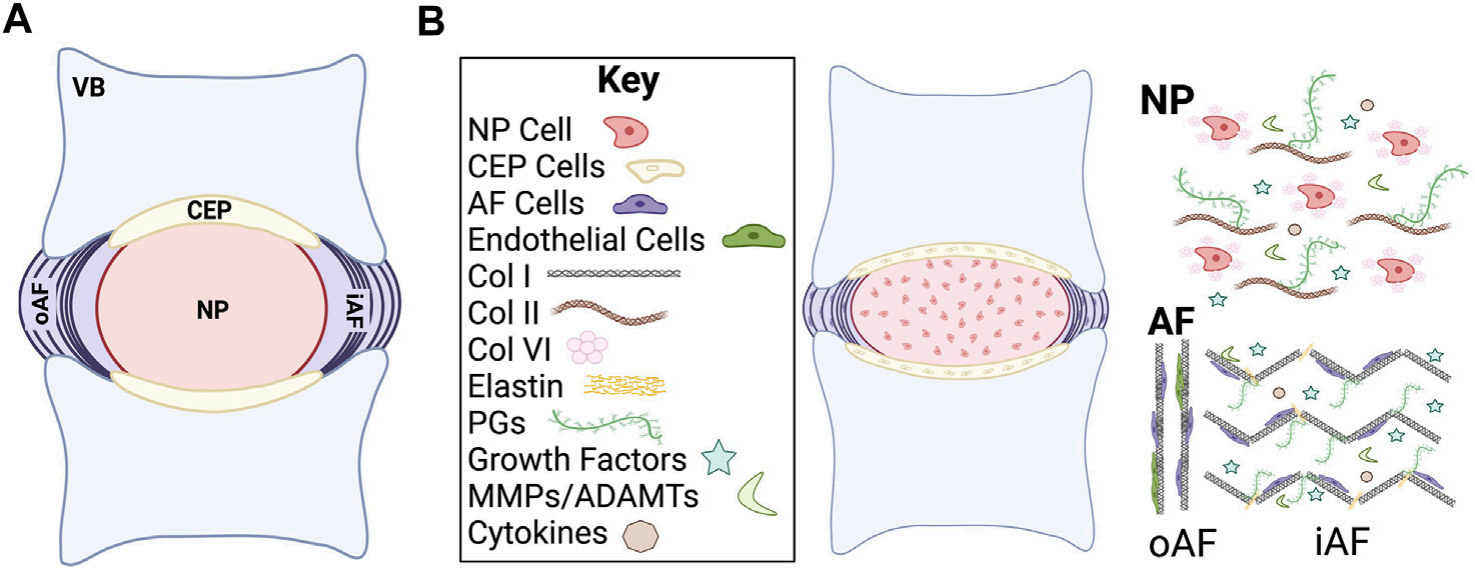
Schematic of intervertebral disc (IVD) structure. **(A)** Midsagittal cut plane of the IVD. **(B)** Overview of general cellular and chemical composition of the IVD. VB, vertebral body; CEP, cartilage endplate; NP, nucleus pulposus; oAF, outer annulus fibrosus; iAF, inner annulus fibrosus; Col, collagen; PGs, proteoglycans; MMPs, matrix metalloproteases; ADAMTs, a disintegrin and metalloproteinase with thrombospondin motifs.

The IVD is comprised of three substructures: the nucleus pulposus (NP), annulus fibrosus (AF), and two endplates of hyaline cartilage on the superior and inferior sides of the disc referred to as the cartilaginous endplates (CEP) (Figure 1A). Like most tissues, the precursors to the IVD are highly vascularized during early stages of formation, but most of these blood vessels disappear following early development, leaving the IVD as the largest avascular structure in the body (Humzah and Soames, 1988; Eyre et al., 1991; Christ and Wilting, 1992; Rodrigues-Pinto et al., 2014). As such, the IVD relies on passive diffusion and fluid convection from the vertebral bone through the CEP for nutrient consumption and waste removal (Urban et al., 1977, 1982; Roberts et al., 1989; Urban et al., 2004; Huang et al., 2014). Nutrients take two primary pathways; the blood vessels that surround the outer AF and the embedded blood vessels that terminate at the bony endplate next to the cartilaginous endplates (Holm et al., 1981; Adams and Hutton, 1986; Grunhagen et al., 2006). Passive diffusion is believed to be the leading mode of transport for smaller molecules such as glucose, lactic acid, and oxygen and is influenced by concentration gradients (Rajasekaran et al., 2004; Magnier et al., 2009). Fluid convection transports larger molecules such as growth factors, proteins, enzymes, and hormones and is influenced heavily by hydraulic pressure gradients (Maroudas, 1975; De Geer, 2018).

The IVD is primarily composed of extracellular matrix (ECM) molecules, including multiple types of collagens and proteoglycans (Roughley et al., 2011; Sivakamasundari and Lufkin, 2012; Sivan et al., 2014a) (Figure 1B). The proteoglycans are highly hydrophilic, which allows the IVD to maintain consistent osmotic pressure (Iozzo and Schaefer, 2015; Dou et al., 2021). Collagen type and spatial distribution vary depending on the IVD region but type I and II collagen are the predominant species. Type I collagen and type II collagen are both fibrillar collagens that undergo significant changes in composition and organization as the IVD develops into a load bearing structure, which allow the IVD to withstand internal and external forces that are applied to the spine. The ECM plays a substantial role both in providing physical structural support to the IVD as well as in regulating biological and cellular signals during development.

The main function of the NP, the inner core of the IVD, is to resist compression of the spine and to redistribute the applied load to the spine (Buckwalter, 1995; Lundon and Bolton, 2001). The healthy NP is primarily comprised of highly gelatinous and hydrophilic material that is a combination of negatively charged proteoglycans, collagen, and non-collagenous proteins. Proteoglycans are made up of core proteins that are covalently attached to one or more types of highly anionic glycosaminoglycans (GAGs). The most abundant proteoglycan in the NP is aggrecan, but it should also be noted that decorin, biglycan, fibromodulin, and versican have also been observed (Sztrolovics et al., 1999; Melrose et al., 2001). The NP has a higher concentration of type II collagen as compared to type I collagen. Other key ECM proteins such as elastin, fibronectin, and laminin are also found in abundance in the NP and confer important physical and biological functionality (Yu et al., 2002; Chen et al., 2009).

The main cell types in the NP are categorized as notochordal and chondrocyte-like large vacuolated cells (Roughley, 2004). NP cells have a large diameter (30–40 µm diameter) and exist in the disc as highly organized interconnected cell clusters that allow for cell-cell communication (Pattappa et al., 2012; Hwang et al., 2014). From early development, NP cells have a highly organized cytoskeleton network that is composed of F-actin, microtubules, vimentin, and cytokeratin intermediate proteins (Li et al., 2008). A strong cytoskeleton network plays an integral role in the ability of the NP to withstand the compressive mechanical load and stress. In contrast to developing NP cells, mature NP cells are described as chondrocyte-like, are smaller (∼10 µm diameter), and do not contain intracellular vacuoles (Hunter et al., 2003). In addition to the loss of cellularity in the NP, the water and proteoglycan content also decreases through ageing, leading to a reduction in disc height and nucleus pressurization over time (Bridgen et al., 2013). The transition from notochordal cells to chondrocyte-like cells is currently debated, with some evidence that notochordal cells are completely replaced with chondrocyte-like cells in certain model systems while other studies show retention of a population of notochordal cells past early developmental stages (Minogue et al., 2010; Risbud et al., 2010). Since notochordal cells are highly synthetic, particularly for hydrophilic ECM molecules like aggrecan core protein and GAGs, either complete or gradual loss of this cell population in the IVD likely contributes to increased risk of degeneration over time (Risbud and Shapiro, 2011).

The AF provides support to the gelatinous structure of the NP by encasing the disc in lamellar-structured rings that are mainly composed of organized collagen fibers and some fibroblast-like cells (Sivakamasundari and Lufkin, 2012; Colombier et al., 2014b). These lamellae resist external forces exerted on the IVD components (García-Cosamalón et al., 2010). There are two main regions of the AF, the inner and outer. The inner AF is primarily composed of type II collagen, proteoglycans, and poorly organized ECM. In comparison to the inner AF, the outer AF is highly organized and rich in type I collagen (Colombier et al., 2014a). The cells of the inner AF are morphologically rounder in shape while the cells in the outer AF are more elongated (Williams et al., 2019). This difference in organization is tied to the function since the outer layer provides more resistance to external tension. The cells of the AF are specifically derived from a population of Scleraxis (*Scx*)+/SRY-related HMG-box (*Sox*)9+ progenitors that are originally unorganized but eventually align into the lamellar pattern as determined by actin stress fiber networks (Hayes et al., 1999; Sugimoto et al., 2013). This cellular alignment is thought to be critical for the organized formation of the collagen matrix, since the patterning lays the template for the lamellae structure (Torre et al., 2019). AF cells are generally characterized as fibroblast-like elongated cells that are organized in concentric circles around the NP (Peacock, 1951).

Two CEPs are located on the cranial and caudal ends of the IVD. CEPs are comprised of subchondral bone and hyaline cartilage and are the linkage points between the vertebral bone and the AF (Sivakamasundari and Lufkin, 2012). CEPs are rich in type II collagen and proteoglycans with a water content of 50–60%, with chondrocytes making up most of the resident cell population (Roberts et al., 1989). The CEPs allow for the transfer of load that is applied to the IVD and play an important role in the flow of nutrients/waste in and out of the IVD.

### Intervertebral Disc Development

During gastrulation, the blastula reorganizes from the single layer of cells into layers of cells that can begin the process of differentiating into germ layers: ectoderm, endoderm, and mesoderm. IVD components arise from the chordamesoderm or paraxial mesoderm. The chordamesoderm gives rise to the notochord and, ultimately, the NP. The paraxial mesoderm gives rise to the sclerotome that forms the vertebrae, CEP, and AF (Colombier et al., 2014b).

#### Nucleus Pulposus

**FIGURE 2:**
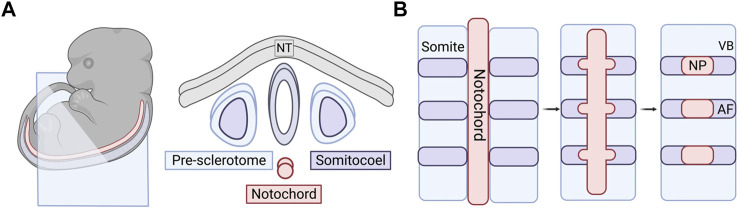
Embryonic and early formation of the intervertebral disc (IVD). **(A)** Embryonic tissues and cell sources that play a role in forming the spinal column. Right panel is a cross-sectional view of the blue cut plane indicated in the embryo in the left panel. **(B)** Sclerotome and somite condensation in formation of vertebral bodies and the annulus fibrosus. Notochord condenses into distinct bulges along the long axis where the NP will form. The AF tissue surrounds the forming NP over time and the VBs section off in between the individual IVDs. NT, neural tube; IVD, intervertebral disc; VB, vertebral body; AF, annulus fibrosus; NC, notochord; NP, nucleus pulposus.

The nucleus pulposus is composed of cells that arise from the embryonic notochord, a transient midline structure that serves as the primitive axial skeleton in the developing embryo (Figure 2A). Importantly, the notochord is the main signaling center that directs the patterning of the surrounding tissues during early development (Pourquié et al., 1993; Fouquet et al., 1997; Stemple, 2005). Forkhead box A2 (*FoxA2*), Brachyury (*T*), and Notochord homolog (*Noto*) are all proven to be essential for regulating the development of the notochord (Vujovic et al., 2006; Maier et al., 2013). In addition to T-brachyury, expression of Sonic hedgehog (*Shh*) and hepatocyte nuclear factor (*HNF*)-3β (also known as *FoxA2*) is important for successful differentiation and survival of notochordal cells (Risbud and Shapiro, 2011). T-brachyury is mainly restricted to the notochord and tailbud and has been shown to affect the development of the tail and vertebrae (Wilkinson et al., 1990; Pattappa et al., 2012). The transcription factor T-brachyury and adherens junction protein N-cadherin are highly expressed NP cells during early development as compared to mature NP cells or NP cells in a degenerated disc environment (Minogue et al., 2010). *FoxA2* primarily affects the development of the node, notochord, and floor plate (Ang and Rossant, 1994). The unique developmental origin of the NP has been confirmed by multiple labs through lineage tracing and mouse model studies as described below (Walmsley, 1953; Hunter et al., 2003; Choi et al., 2008; McCann et al., 2012; Sivakamasundari and Lufkin, 2012; Alkhatib et al., 2018).

SHH protein plays a critical role in the patterning of the anterior-posterior axis during embryonic development (Harfe et al., 2004). Using a tamoxifen-inducible *Shh*-cre mouse model, Choi et al. produced a fate map of SHH-expressing cells in the axial skeleton to track notochordal cells throughout their development (Choi et al., 2008). Through the activation of R26R:eYFP in the notochord, they were able to track the notochord cells via YFP fluorescence and concluded that the majority of the cells become localized to the nucleus pulposi. There are a few notochordal cells that have been found in the spinal column outside of the NP in lineage tracing studies, but it is unclear as to whether this is a result of aberrant localization during development. It has also been conjectured that these minor populations give rise to chordomas later in life. Choi & Harfe expanded on this work by removing the hedgehog signaling at different stages of development (Choi and Harfe, 2011). When signaling was removed from all SHH-expressing cells, the NP was smaller in the mutant mice when compared to the control. The AF also lost the concentric rings, which the authors speculated was a result of the lack of NP development. As the notochord develops, bulges form along the long axis where the IVDs will eventually form the NP (Figure 2B) (Smith et al., 2011; Tomaszewski et al., 2015). In the mutant mice, the notochord was not only thinner, but these bulges never developed. The notochordal cells were instead found all along the length of the entire spinal column rather than localized to disc regions. They also temporally knocked out SHH protein signaling before and after the formation of the notochord sheath. They found that signaling is required before the sheath formation for proper patterning of the spinal column but is not required later in development to maintain structure (Alkhatib et al., 2018).

In a separate study, McCann et al. developed a *Noto*-cre mouse model to target node and posterior notochord formation during gastrulation (McCann et al., 2012). By examining staining patterns of β-galactosidase and cytokeratin-8 as the embryos developed, they were able to trace the notochordal-derived cells, which independently confirmed that notochordal cells do indeed give rise to the NP. McCann et al. also demonstrated that small chondrocyte-like NP cells were derived from the notochord and were phenotypically distinct when compared to chondrocytes from articular cartilage (Clouet et al., 2009; Minogue et al., 2010). Genes such as Danforth’s short tail (*Sd*), *Shh*, smoothened (*Smo*), *Sox5/Sox6*, and TSC Complex Subunit 1 (*TSC1*) were essential for NP development (Lane and Birkenmeier, 1993; Alfred et al., 1997; Choi and Harfe, 2011). In addition to their contribution to the formation of the NP, notochordal cells also aid in the synthesis of ECM during the formation of the spinal column through the stimulation of growth factors (Pattappa et al., 2012).

#### Annulus Fibrosus

The AF, along with the vertebrae, CEPs, tendons, and ligaments are derived from the sclerotome of the somites (Williams et al., 2019; Bagnat and Gray, 2020). Prior to the specification of the sclerotome, the somites are derived from the paraxial mesoderm through the inhibition of bone morphogenetic protein (BMP) signaling in the lateral plate mesoderm by follistatin, an activin-binding protein, working in tandem with Noggin (Stafford et al., 2014). After the specification and formation of the sclerotome, it undergoes organization and resegmentation where the cells at the caudal boundary of the von Ebner’s fissure, the furrow that divides the rostral and caudal halves of the somite, will develop into the AF or the vertebral body through condensation of the cells (Christ and Wilting, 1992; Christ et al., 2007; Alkhatib et al., 2018) (Figure 2B). The AF will further differentiate into the inner AF, a region that exhibits characteristics of fibrous tissue and cartilage, and outer AF, a primarily fibrous region (Van den Akker et al., 2017).

There are many signaling pathways involved in the determination of the AF. Mesenchyme forkhead 1 (*Mfh1*) and transforming growth factor (*TGF*)-β1 are known to play vital roles (Sivakamasundari and Lufkin, 2012). Paired box (*Pax*) 1 and *Pax9* deletions can also affect the sclerotome cell proliferation, leading to a lack of vertebral bodies, proximal portions of the ribs, and IVD (Wallin et al., 1994; Peters et al., 1999; Baffi et al., 2004; Jin, 2011; Mundy et al., 2011). Initial *Pax1* and *Pax9* expression is seen throughout the AF, but expression eventually becomes enriched in the caudal region later in development (Williams et al., 2019). Additional mouse studies have shown that Indian hedgehog (*Ihh*), *TSC1*, and Wnt/βcat are required for AF formation (Maeda et al., 2007; Kondo et al., 2011; Yang et al., 2017; Silva and Holguin, 2020). TGF-β1 plays a pivotal role in the development and growth of the spine, particularly during the differentiation of the sclerotome into cells that eventually form the AF (Gantenbein-Ritter et al., 2011; Stoyanov et al., 2011; Leung et al., 2014). Genes and signaling pathways that play an important role in spinal column patterning and IVD formation are summarized in Table 1.

**TABLE 1:** Summary of genes and signaling pathways that play an important role in intervertebral disc development.

Gene	Model	Development	Degeneration	Citation
*ApoE*	KO	Decreased GAG in NP cells	Early IVD degeneration	Zhang et al. (2013)
*Bapx1*	KO	Skeletal dysplasia in the vertebral column; Struncated axial skeleton		Chatterjee et al. (2014)
*Bgn*	KO		Accelerated IVD degeneration	(Furukawa et al., 2009)
*Casp3*	KO	Smaller IVDs and body size, higher IVD cell density	Accelerated age-related IVD degeneration	(Ohnishi et al., 2019)
*CCN2*	cKO	Disrupted IVD formation	Accelerated age-related degeneration	(Bedore et al., 2013)
*Col1a1*	KO	Mechanical properties of discs were significantly inferior		(Sarver and Elliott, 2004)
*Col2a1*	KO	Less GAG in IVD	Mild disc degeneration	(Sahlman et al., 2001)
*Col9a1*	KO		Accelerated IVD degeneration, NP shrinkage, AF fissures	(Kimura et al., 1996)
*Ext1*	cKO	Missing IVDs and aberrant organization		Mundy et al. (2011)
*Foxa1/Foxa2*	cKO	Severe NP deformation, missing tail IVD		Maier et al. (2013)
*GDF-5*	KO	Loss of normal AF structure, disorganized NP, decreased PG content		(Li et al., 2004)
*Has2*	KO	Changes in NP cellular organization, early mortality		Roughley et al. (2011)
*HIP-1α*	cKO		Accelerated IVD degeneration	(Meng et al., 2018)
*IFT80*	cKO	Disrupted structure, cilia loss		Li et al. (2020)
*Ihh*	cKO	Enlarged NP, no AF		Maeda et al. (2007)
*IL-1*	KO		Increased degeneration in AF	Gorth et al. (2019)
*Jun*	cKO	NP hypocellularity, defective IVD formation		Behrens et al. (2003)
*NF-κB*	KO		Increased PG synthesis and reduced disc cellularity loss	Nasto et al. (2012)
*Pax1*	KO	IVD absent		Wallin et al. (1994)
*Progranulin*	KO		Early onset of IVD degeneration	Zhao et al. (2015)
*Runx2*	cKO	Vacuolated cells in NP		(Liao et al., 2019)
*Sd*	KO	Absence of NP and reduced vertebrae number		Lane and Birkenmeier (1993); Alfred et al. (1997)
*Shh*	cKO	Absence of NP		Choi and Harfe (2011)
*Skt*	KO	Dislocation of NP and kinky tail		Semba et al. (2006)
*Smo*	cKO	Aberrant NP formation		Choi and Harfe (2011)
*Sox5/Sox6*	KO	Absence of NP		Smits and Lefebvre (2003)
*Sox9*	cKO		AF and NP ECM remodeling, progressive IVD degeneration	Tsingas et al. (2020)
*TSC-1*	cKO	NP and AF absent		Yang et al. (2017)
*Tgfbr2*	cKO	Incomplete or missing IVD	Loss of matrix in inner AF, IVD maintenance defects	Baffi et al. (2004); Jin (2011)
*Wnt/βcat*	cKO	Severe AF deterioration	Increased IVD degeneration	Kondo et al. (2011); Silva and Holguin (2020)

AF, annulus fibrosus; NP, nucleus pulposus; GAG, glycosaminoglycan; IVD, intervertebral disc; ECM, extracellular matrix; PG, proteoglycan; KO, knockout; cKO, conditional knockout.

### Intervertebral Disc Degeneration

Intervertebral disc degeneration (IDD) is a cell-mediated response to the functional and structural failure of the disc (Adams and Roughley, 2006). Disc degeneration is caused by multiple factors and is associated with ageing, mechanical stress, genetic factors, and nutritional disorders. Mechanical stress can arise from hypermobility and overloading of the disc in the early stages of IDD, which is then generally followed by limited motion and hypomobility in the later stages (Stokes and Iatridis, 2004). Other physical factors that contribute to IDD include obesity, high BMI, bad posture, and smoking (Fogelholm and Alho, 2001; Hangai et al., 2008; Elmasry et al., 2015; Sheng et al., 2017). IDD can lead to many related and often progressive medical conditions as a result of spinal instability, disc herniation, and nerve impingement such as spondylosis, which refers to degenerative and osteoarthritic changes to the discs and vertebral bones, and sciatica, where pressure put on the sciatic nerve root by a herniated disc or bone spur can cause discomfort, pain, and limb weakness (Mulleman et al., 2006; Ferrara, 2012).

Although IDD is a multifactorial disease, recent studies have shown that genetic factors play a critical role in the onset and progression of IDD (Feng et al., 2016). Population studies have indicated polymorphisms in genes such as *COL1A1*, *COL9A2*, *COL11A1*, Aggrecan, and IL-1 may play a role in disc degeneration (Annunen et al., 1999; Kawaguchi et al., 1999; Solovieva et al., 2004; Tilkeridis et al., 2005; Mio et al., 2007; Janeczko et al., 2014; Yang et al., 2019). Due to limitations in collecting human patient samples, very few genome-wide association studies (GWAS) in humans have been completed (Urban and Fairbank, 2020). Human GWAS meta-analyses have found novel candidate genes that, when mutated, may be associated with disc degeneration, including *PARK2* (Williams et al., 2013) and *CHST3* (Song et al., 2013). The GWAS analysis that identified *PARK2* involved 4,600 northern European individuals with IDD and suggested that methylation of the *PARK2* promoter may induce IDD. The analysis that identified *CHST3* as a candidate gene in disc degeneration involved a total of 32,642 human subjects of Southern Chinese descent, with 4,083 individuals with lumbar disc degeneration and 28,599 controls. This study found that mutations in *CHST3* altered synthesis of ACAN, one of the crucial proteoglycans responsible for disc hydration. Meta-GWAS analysis of 158,000 individuals of European descent identified 3 novel loci; rs12310519 in *SOX5*, rs4384683 in *CCDC26/GSDMC*, and rs4384683 in *DCC*, that may be associated with chronic back pain (Suri et al., 2018). In addition to the three above mentioned loci, a follow up study by Freidin *et al.* also found that mutations in *SPOCK2* and *CHST3* potentially contribute to disc generation and general back pain in a study of 509,000 European individuals (Freidin et al., 2019). However, there is debate as to whether the *SOX5*, *CCDC26/GSDMC*, and *DCC* loci are associated with back pain based on another study of Caucasian individuals in Norway (Lie et al., 2022).

Reduction and long-term loss of nutrition also appear to be key factors responsible for disc cell changes (Buckwalter, 1995; Urban et al., 2004). Blood vessels in the disc margins supply the disc with essential nutrients and substrates (Eyre et al., 1991). Ageing and prolonged mechanical load can damage the CEPs, impairing nutrient delivery and waste removal from the IVD, resulting in disc tissue breakdown (Boos et al., 2002). Increasing age and degeneration also result in disorganization of the disc morphology. This includes disorganization of the annular lamellae with decreased proteoglycan content, bifurcation, and interdigitation (Lai et al., 2015). While increased cell proliferation as a response to damage and stress has been observed (Johnson et al., 2001), there seems to be a concomitant increased senescence and apoptosis (Gruber and Hanley, 1998; Roberts et al., 2006).

ECM homeostasis is maintained through catabolic and anabolic regulation and is essential for the structural integrity of the IVD (Moon et al., 2014). In IDD, there is increased expression of matrix-degrading proteins such as matrix metalloproteinases (MMPs) and a disintegrin and metalloproteinase with thrombospondin motifs (ADAMTs) resulting in deregulation of the normal homeostatic mechanism (Le Maitre et al., 2007). The loss of proteoglycans is a major biochemical change observed in IDD. As aggrecan becomes degraded, associated GAGs are lost, which leads to loss of hydration in the NP (Sivan et al., 2014b; Wei et al., 2019). This reduction of NP hydration results in loss of disc height and disc bulging (Kumaresan et al., 2003). Degenerating discs also show loss of collagen fibers and increase in fibronectin (Hollander et al., 1996; Oegema et al., 2000).

Oxidative inflammation is also thought to be a major cause of molecular damage through various stressors (McGarry et al., 2018). While IVDs reside in a low oxygen tension environment, reactive oxygen species (ROS) are generated through oxidative phosphorylation (Ali et al., 2008). ROSs damage lipids, DNA, and proteins, and affect the structural and functional homeostasis of the disc (Feng C. et al., 2017).

Several animal models, such as sand rats and Chinese hamsters exhibit naturally occurring IDD (Silberberg and Gerritsen, 1976; Adler et al., 1983; Moskowitz et al., 1990), but in consideration of consistency and time-constraints in IVD research, multiple disc degeneration models have been generated using physical, chemical, or genetic modification (Singh et al., 2005). Specifically, SM/J mice, known for their poor regenerative capacities, displayed early spontaneous onset IDD that was marked by early onset cellular and matrix changes that were consistent with human IDD, such as loss of NP/AF boundary, formation of small clusters in the NP, and chondrocyte-like cells surrounded by proteoglycan-rich matrix (Choi et al., 2018). Furthermore, in a comparative study of SM/J mice and LG/J mice, a strain known for its super tissue healing capacity, Zhang et al. reported that changes in the NP affected ion regulation within this disc region, which led to dysregulated cellular homeostasis and fibrotic changes to the tissue (Zhang et al., 2018).

Physical methods to induce degeneration have included protocols such as forelimb amputations to force bipedalism, annulotomies, and endplate injury (Yamada, 1962; Stern and Coulson, 1976; Lipson and Muir, 1981; Higuchi et al., 1983; Holm et al., 2004). Chemical methods include chemonucleolysis, fibronectin fragment injections, and smoking (Bradford et al., 1983; Simmons et al., 1984; Holm and Nachemson, 1988; Iwahashi et al., 2002; Greg Anderson et al., 2003). One of the more commonly used methods to induce disc degeneration in recent studies has been the needle puncture injury method in both *in vivo* and *ex vivo* small and large animal models, where the IVD is punctured using needles of various gauges (Korecki et al., 2008; Michalek et al., 2010; Martin et al., 2013; Piazza et al., 2018). The severity of degeneration can be varied based on the location and depth of the puncture and size of the needle used (Moriguchi et al., 2018; Yang et al., 2018; Qian et al., 2019). The caudal IVD is frequently targeted for this method due to the ease of access as the caudal spine lacks transverse and posterior processes and does not encase the spinal cord. While the needle puncture injury method provides reliable and consistent experimental disc degeneration models, findings from these studies need to be carefully interpreted and considered in a context dependent manner as the animal caudal spine differs in anatomy, mechanical load, and degrees of motion as compared to the human spine.

Non-rodent animal models for IVD include rabbits, which display a similar disc morphology to humans and have been used to study degeneration as well as potential therapeutics (Kroeber et al., 2002; Liu et al., 2011; Calvo-Echenique et al., 2018). Multiple larger animal models have also been established including canine, ovine, caprine, camelid, and porcine models (Reid et al., 2002; Yoon et al., 2008; Bergknut et al., 2012; Stolworthy et al., 2015; Gullbrand et al., 2017a). Due to their similarity to humans, non-human primates have been used as models for IVD research and therapeutics. Although they are usually quadrupedal, primates such as baboons and macaques spend significant time in erect positions and display spontaneous disc degeneration (Lauerman et al., 1992; Platenberg et al., 2001; Gal, 2002; Nuckley et al., 2008). Rhesus monkeys have been utilized as they display age-related IDD with comparable histologic pathology to human disc degeneration (Bailey et al., 2014). Baboons and rhesus monkeys have also been used to analyze the efficacy of IVD prostheses and autograft surgeries (Luk et al., 1997; Cunningham et al., 2002; Luk et al., 2003; McAfee et al., 2003). Non-human primates and large animal models are particularly useful models as their discs are similar in size and morphology to humans. Primates also face biomechanical stress from an upright position that cannot be replicated in quadrupedal animal models. However, their usage involves extensive ethical considerations, and the high costs and practical requirements can restrict their use in many studies.

### Pain Management in Current IDD Therapies

Although there are currently no cures for IDD and back pain, there are multiple chemical treatments that are used to manage IDD and discogenic pain. The initial standard approach to treating lower back pain is a 4–6-weeks conservative therapy course involving analgesics, NSAIDs (Non-steroidal anti-inflammatory drugs), and physical therapy (Masala et al., 2019). If there is no improvement or relief from this treatment, percutaneous procedures are utilized. Percutaneous procedures serve as an intermediate step between conservative therapy and decompression and surgical options (Masala et al., 2019).

There are a variety of percutaneous procedures that can be classified into three categories: annuloplasty, percutaneous disc decompression, and endoscopic percutaneous discectomy. Annuloplasties are procedures aimed at repairing the disc annulus (Kapural and Houra, 2012). Intradiscal electrothermal therapy (IDET), a form of annuloplasty, is a minimally invasive surgical procedure where heat is applied to the AF through a catheter, preventing future ruptures through the change in collagen structure and burning of nociceptors to decrease the level of pain (Saal and Saal, 2000a; Saal and Saal, 2002b). Radiofrequency is also frequently utilized to treat discogenic pain with methods such as radiofrequency annuloplasty (RFA), which is a minimally invasive method that delivers radiofrequency thermal energy to the disc to denervate the AF and provide pain relief (Gelalis et al., 2019). Intradiscal radiofrequency thermocoagulation (IRFT) involves the direct application of radiofrequency energy to the center of the disc (Troussier et al., 1995). Another annuloplasty method is the intradiscal biacuplasty, which involves placing two radiofrequency electrodes on the posterolateral sides of the annulus fibrosus (Kapural and Mekhail, 2007).

Despite the theoretical benefits of these treatments and their extensive use, it appears that they have little clinical advantage over sham procedures (Kepler et al., 2011). One clinical study involving 57 patients suffering from chronic discogenic lower back pain (CDLBP) observed the effects of IDET (38 patients) or a sham procedure (19 patients) and reported no significant improvement in either group (Freeman et al., 2005). Another study involving 28 CDLBP patients compared the clinical outcomes of IRFT (13 patients) to a placebo treatment (15 patients) and found that IRFT was not effective in reducing CDLBP (Barendse et al., 2001). However, it should be noted that both of these trials were conducted on a small sample size and further large-scale clinical trials are needed to provide more information on the clinical relevance of these treatments.

Other percutaneous procedures targeting herniation and discogenic pain include endoscopic discectomy and disc decompression that includes laser discectomy, plasma discectomy, and mechanical disc decompression (Raj, 2008). Endoscopic discectomy, which endoscopically removes a portion of the NP, is a good therapeutic treatment for discogenic pain and disc protrusion (Xu G. et al., 2020). Percutaneous lumbar laser discectomy involves using laser energy to vaporize a small volume of the NP resulting in reduced pressure; however, there is limited evidence of the clinical benefits (Singh et al., 2013). Currently there are commercial decompression systems such as the Dekompressor or high RPM devices available for percutaneous discectomies. The device is a high rotation device that extracts nuclear material through a cannula. A review found limited evidence for the use of the Dekompressor; however, due to the small number of both randomized or observational trials, further research has to be conducted on the method (Manchikanti et al., 2013). Overall, despite their widespread use over the years, it appears that multiple percutaneous disc procedures do not have significant clinical relevance in the treatment of IDD and discogenic pain.

Chemonucleolysis is a form of non-surgical treatment for discogenic pain and herniated discs (Alexander, 1986), which involves the administration of a proteolytic enzyme like chymopapain into the IVD to alleviate fibrotic tissue damage and reduce disc bulging (Thomas, 1956). Unfortunately, the long-term effects of chemonucleolysis includes lower back pain partially due to depolymerization of the overall NP structure and loss of water content. Due to these adverse effects, the technique is no longer used in the United States and Europe. A newer version of this treatment uses a more specific GAG-degrading enzyme condoliase (Chiba et al., 2018; Matsuyama and Chiba, 2019; Muramatsu et al., 2020).

As conservative therapy and percutaneous procedures are quite palliative in nature and may restrict functionality, surgery is utilized to help alleviate discogenic pain and with the hope of providing better mobility and function. Surgical treatments may include IVD excision, spinal fusion, or artificial disc replacement (Bowles and Setton, 2017). Spinal fusion is a commonly used surgical procedure to treat IDD that involves joining two or more vertebrae using bone grafting (Tehranzadeh et al., 2005). Spinal fusion has many advantages including elimination of motion at the degenerated disc, allowing for better decompression and elimination of instability. Currently, bone grafts are obtained from the patient themselves (autograft), a consenting donor (allograft), or using artificial bone substitutes. To eliminate the morbidity relating to autografts and allografts, there has been extensive development of artificial substitutes (Gupta et al., 2015). Artificial bone substitutes have been created from natural materials such as demineralized bone matrix substitutes and ceramic substitutes, originally obtained from corals (Girardi and Cammisa, 2003; Chen et al., 2005). Due to the recent advances in tissue engineering, there is also promise of using biomaterials such as hydrogels and nanofiber scaffolds as synthetic bone substitutes. The possibility of being able to release controlled growth factors from the biomaterial during spinal fusion also makes biomaterials an attractive solution (Gupta et al., 2015).

Despite hundreds of thousands of individuals receiving spinal fusion surgery annually, there are various factors that bring into question the clinical significance and efficacy of the procedure. A study observed that for nonradicular back pain, fusion surgery was no more effective than intensive rehabilitation (Chou et al., 2009). An analysis of patient satisfaction indicated that on average 68% of patients were satisfied after spinal fusion surgery with frequently reported complications including pseudoarthrosis (14%) and chronic pain at the graft site (9%) (Turner et al., 1992). A large clinical study in South Korea of 629 patients who underwent spinal fusion also found that post-operative patient satisfaction was around 71%, with unsatisfied patients reporting the persistence of chronic pain after the surgery (Kong et al., 2010). Analysis of a cohort of 874 patients who underwent lumbar spinal fusion found that at 24 months post-surgery, an alarming 44.4% of patients had been prescribed opioids for continued pain management and 37.8% of patients were receiving mental health treatment for stresses related surgery and back pain (McMillan et al., 2021). Additional studies have shown that spinal fusion may result in degeneration of adjacent discs and lead to further discogenic pain, herniation, stenosis or spondylosis, suggesting that improvements in surgical interventions for back pain are necessary (Ghiselli et al., 2004).

As spinal fusion surgery may limit mobility, artificial discs or cervical disc replacement (CDR) devices have emerged as an alternative as they preserve range of motion (Greg Anderson et al., 2003). As of 2020, there are 15 artificial discs actively used in global markets with 7 of them approved for use in the United States (Turel et al., 2017; Nunley et al., 2018). Clinical studies have demonstrated that artificial disc replacements are a viable alternative to spinal fusion or percutaneous procedures and are superior in terms of neurological success and range of motion (Heller et al., 2009; Gao et al., 2015). Studies have also shown that the clinical outcomes may differ among various CDR devices, requiring surgeons to tailor the device choice to specific patient profiles (Wahood et al., 2020). Complications from disc replacement can include incomplete decompression, malposition, infection, and vertebral body fracture (Steinberger and Qureshi, 2020). Despite their benefits, due to the technical challenges of the surgical procedure, the high cost, and outcomes similar to spinal fusion, total IVD disc replacements are not yet widely used (Phillips et al., 2015).

### Development of Regenerative Strategies and Therapeutics

Due to limitations of current treatments, there has been emerging interest in the treatment of IDD using cell-based and biomaterial engineering therapies as summarized below. Preclinical and clinical studies demonstrate potential for the treatment of disc degeneration using cell-based therapies (Sakai and Schol, 2017; Urits et al., 2019). Currently ongoing and recently completed clinical trials are summarized in Table 2. Many clinical trials have focused on injection of a combination of growth factors, biomimetic materials, and cultured cells with varied success. However, due to small sample sizes and lack of comparative studies, extensive research needs to be conducted before the widespread introduction of these treatments (Meisel et al., 2019). Furthermore, as cell-based therapies primarily involve implantation of stem cells, the source of these cells can lead to ethical issues and treatment scrutiny. Currently, most clinical trials utilize autologous stem cells that are expanded in an *in vitro* tissue culture setting prior to use for treatment. However, as autologous stem cells are not always available for harvesting dependent on disease severity and presence of comorbidities in patients, some trials are exploring the use of allogeneic cells. This requires additional testing for immunologic and inflammatory responses, potential transmittable diseases, as well as additional consenting steps from the donors prior to implantation. The ethical and legal considerations for stem cell treatments and clinical trials are also dependent on each individual country’s healthcare and biomedical legislations, further complicating widespread implementation of cell-based therapeutics (Dhar and Hsi-En Ho, 2009). Further complicating the strategy of directing cellular differentiation into healthy IVD tissue is the unique developmental origin of the NP. As discussed previously, unlike most of the surrounding tissue that is of mesenchymal origin, the NP arises from the notochord, which is a transient structure. In some animals, especially rodents, there is evidence of molecularly distinct notochordal cells that persist in the NP well past the early developmental stages. However, this cell population disappears at early neonatal stages in humans, and thus, there is no good natural cellular depot from which these cells can be harvested and cultured in human patients. Therefore, a significant challenge in cell-based NP regeneration therapies is defining the unique cellular genotype and phenotype of the progenitor notochordal cells and recapitulating this distinct cellular profile in cells of non-notochordal origin.

**TABLE 2:** Summary of clinical trials for IVD treatment.

Trial ID	Start Year	Status	Location	Summary	Enrollment*	References
NCT01124006	2010	Completed in 2014	United States	Intradiscal injection of recombinant human GDF-5 in early IDD. Showed improvement in certain parameters	24	
NCT01158924	2010	Completed in 2014	Australia	Intradiscal injection of recombinant human GDF-5 in early IDD.	40	
NCT01182337	2010	Completed in 2014	South Korea	Intradiscal injection of recombinant human GDF-5 in early IDD.	31	
NCT01290367	2011	Completed in 2015	United States	Intradiscal injection of 2 doses of allogenic adult MPCs in hyaluronic acid	100	
NCT01513694	2010	Completed in 2017	Spain	Implantation of tricalcium phosphate embedded MSCs. VAS and ODI scores improved and 80% of patients achieved lumbar fusion. The procedure is feasible, safe, and potentially effective	15	Blanco et al. (2019)
NCT01771471	2012	Terminated	United States	Intradiscal delivery of juvenile chondrocytes in fibrin carrier	44	
NCT01860417	2013	Completed in 2017	Spain	Intradiscal injection of allogenic MSCs. Showed significant improvement in algofunctional indices and degeneration	25	Noriega et al. (2017)
NCT02097862	2014	Completed in 2017	United States	Intradiscal injection of ASCs. Patients showed improvements in various parameters	15	Comella et al. (2017)
NCT02205138	2014	Competed in 2021	Belgium	Administration of allogenic osteoblastic cells with ceramic scaffold into lumbar fusion site	38	
NCT02338271	2015	Status unknown	South Korea	Injection of autologous ASCs with hyaluronic acid derivatives	10	
NCT02379689	2014	Status unknown	United States	Intradiscal injection of placental tissue extract	30	
NCT02529566	2013	Unknown	United States	Tissue grafting of human MSCs	100	
NCT03340818	2018	Unknown	United States	Intradiscal injection of autologous BM concentrate	60	
NCT03347708	2018	Ongoing	United States	Intradiscal injection of 2 doses of discogenic cells in Sodium Hyaluronate	60	
NCT03390920	2022	Unknown	United States	Use of amniotic fluid tissue product in IDD	200	
NCT03461458	2018	2021	United States	Intradiscal injection of autologous ASCs	1	
NCT03692221	2019	Ongoing	United States	Image guided percutaneous needle injection of autologous BM-MSCs	24	
NCT03737461	2019	Ongoing	France	Intradiscal injection of allogenic adult BM-MSCs	113	
NCT03827096	2013	Completed in 2016	Czech Republic	Injection of autologous human MSCs with beta-tricalcium phosphate	10	
NCT03912454	2019	Ongoing	United States	Intradiscal injection of BM aspirate concentrate	20	
NCT04414592	2020	Ongoing	China	Grafting human UC- MSCs into degenerated disc	20	
NCT04499105	2017	Ongoing	Indonesia	Implantation of allogenic UC-MSCs	10	
NCT04559295	2018	Ongoing	United States	Intradiscal injection of BM concentrate	80	
NCT04621799	2018	Ongoing	United States	Intra-annular injections of non-autologous fibrin	400	
NCT04727385	2020	Ongoing	France	DXM microgel injection into IVD space	20	
NCT04735185	2022	Unknown	United States	Comparison of intradiscal autologous BM-MSCs and intradiscal steroids	106	
NCT04759105	2020	Ongoing	Italy	Intradiscal injection of autologous BM-MSCs	52	
NCT04816747	2021	Ongoing	Greece	Intradiscal and intra-articular injection of Platelet-rich plasma	50	
NCT04849429	2021	Ongoing	India	Intradiscal injection of Exosome-enriched Platelet-rich Plasma	30	
NCT05011474	2021	Ongoing	South Korea	Implantation of Matrillin-3 pretreated ASC spheroids in lumbar discs	4	
NCT05066334	2021	Ongoing	Italy	Intradiscal injection of autologous BM-MSCs	52	
NCT05146583	2021	Ongoing	United States	Intradiscal injection of autologous BM aspirate	60	

*Estimated enrollment for ongoing studies.

VAS, visual analog scale; ODI, oswestry disability index; MSC, mesenchymal stem cells; ASC, adipose-derived stem cells; BM, bone marrow; UC, umbilical cord; DXM, double cross-linked; MPC, mesenchymal precursor cells; IDD, intervertebral disc degeneration.

Healthy NP tissue generally contains two cell types, depending on age and species: notochordal cells that play an important role in IVD development, growth, and homeostasis, and the nucleopulpocytes that maintain the IVD post development (Clouet et al., 2019). Studies have shown that mouse induced pluripotent stem cells (iPSCs) have the potential to differentiate into NP-like cells (Chen et al., 2013). Based on developmental studies, notochordal cells could be a tool for regenerative medicine. Given their early embryonic origin and limited numbers, they will likely need to be generated from iPSCs or ESCs. One study demonstrated that human iPSCs could be differentiated into notochordal cell-like cells when cultured in the presence of natural NP tissue matrix (Liu et al., 2014; Liu et al., 2015). A separate study focused on the *NOTO* and *NOTO*-related genes’ roles in the commitment of mesendoderm progenitor cells towards a notochordal fate by maintaining an expression of the regulatory factors *FoxA2* and *T-brachyury* (Colombier et al., 2020). Notably, Zhang et al. established a *NOTO*-eGFP reporter in a human pluripotent stem cell (hPSC) line using CRISPR/Cas9-mediated genome editing that was verified by comparing them to two human NP cell transcriptome datasets (Zhang et al., 2020). Currently, human PSCs have limited clinical utilization as rapid growth and safety are of major concern (Hynes et al., 2014; Clouet et al., 2019). However, a new method has been devised to efficiently differentiate human PSCs toward NP-like cells, which may be able to provide a cell source for future regenerative therapies (Tang et al., 2018). Tang et al. developed various chemically defined mediums and growth factor supplementation to direct undifferentiated human PSCs to an NP-genic phenotype through a stepwise differentiation. Early exposure to Activin A and Wnt-3a, followed by varying levels of exposure to BMP4 and FGF2 induced cell differentiation through a mesodermal and notochordal lineage to NP-like cells.

Another potential cell source for cell-based therapy to treat IDD is mesenchymal stem cells, also known as mesenchymal stromal cells (MSCs). MSCs can be derived from robust sources like the bone marrow and adipose tissue, even from adult donors, and escapes much of the ethical and safety scrutiny that ESCs undergo in cell-based therapeutics development (Pittenger et al., 1999; Zuk et al., 2001; Blanco et al., 2010). Animal model studies demonstrated that MSCs injected into the NP can survive for months and proliferate under specific conditions (Hiyama et al., 2008; Henriksson et al., 2009; Shen et al., 2015). Further research has indicated that certain growth factors such as growth differentiation factor (GDF) 5 and GDF6 could induce adipose-derived MSCs (ASCs) to differentiate into NP-like cells (Stoyanov et al., 2011; Clarke et al., 2014; Colombier et al., 2016). Transplantation of MSCs using a hydrogel or colloidal gel carrier also increased the regenerative capabilities (Pereira et al., 2014; Chen et al., 2019; Choi et al., 2020; Wang Y. et al., 2021). In a human trial, injections of autologous expanded bone marrow MSCs into the NP resulted in elevation of water content and rapid improvement of pain (Orozco et al., 2011). While MSCs may be an attractive alternative source to ESCs in cell-based therapies, difficulty in continued proliferation *in vitro*, off-target differentiation into other cell types, fibrotic changes, and cellular senescence are issues that have yet to be fully addressed.

Recent advances in polymer and chemical engineering have led to the rapid development of synthetic and 3D printed bioimplants and hydrogels (Bowles and Setton, 2017; Shi et al., 2020; Li et al., 2021; Yan et al., 2021). Recently developed hydrogels and biomaterials have been summarized in Supplementary Table S1. In regenerative therapeutics, hydrogels and biomaterials are primarily used as scaffolds to support the growth and cell viability of cell-based treatments such as MSC injections. Biomaterial scaffolds can be used in combination with cell-based therapies to support NP regeneration and serve as carriers for cell and vector delivery (Li et al., 2016; Priyadarshani et al., 2016). Nanofiber material such as sponge microspheres (NF-SMS) mimic the fibrous ECM environment whereas materials such as polycaprolactone (PCL) and poly(ether carbonate urethane)urea (PECUU) are used for scaffolds that mimic the biomechanical properties of natural tissue (Zhang et al., 2015; Zhu et al., 2016; Christiani et al., 2019). Synthetic hydrogels, which are 3D network microstructures, show great potential with hydrogels like polyvinyl alcohol (PVA) and polyvinylpyrrolidone (PVP) having similar biomechanical properties to human discs (Katta et al., 2007). Natural materials such as chitin, gelatin, and alginate have also been used to produce hydrogels like chitosan and gelatin methacryloyl (GelMA) that act as scaffolds and promote ECM production (Guo et al., 2014; Li et al., 2017; Yang et al., 2020). Hydrogels and 3D printed implants to replace the damaged NP have shown potential to restore disc height and reduce pain (Schmocker et al., 2016; Perera et al., 2021).

### Omics in IVD Development, Homeostasis, Degeneration, and Therapeutics

**FIGURE 3:**
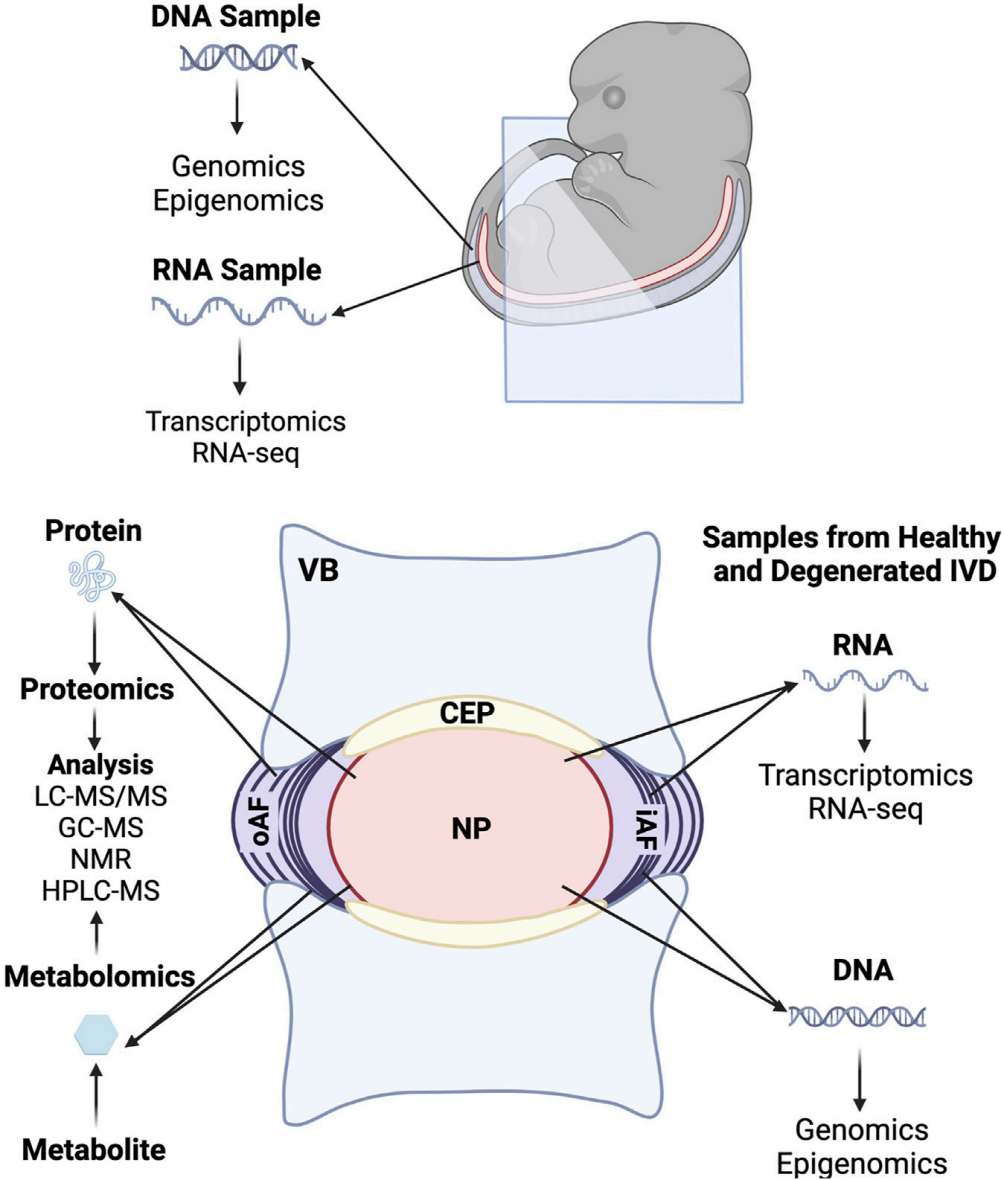
Summary of omics investigations carried out during embryonic development and in the fully formed intervertebral disc.

The advent of many omics techniques such as whole-transcriptomic sequencing (RNA-Seq), proteomics, and metabolomics have revolutionized our understanding of IVD development by allowing researchers to generate global profiles of gene expression and molecular level changes that are occurring at different time points during IVD formation. Omics studies have added much depth to our knowledge of processes involved in IVD development and helped to identify disc specific makers, as well as led to a better understanding of pathways involved in the maintenance and homeostasis of healthy tissue and degenerative changes that occur in IVD pathology. Some of the major omics investigations are discussed in this section and summarized in the omics tables (Figure 3 and Table 3).

**TABLE 3:** Summary of omics studies in IVD research.

Model	Tissue	Method	Findings	References
A. Transcriptomics				
Human	Notochordal cells derived from hESCs-H9 cell line	Pan-genomic high-throughput scRNA-seq	Identified transcription factor regulatory network consisting of notochordal differentiation drivers *PAX6, GDF3, FOXD3, TDGF1, SOX5, LMX1A, LEFTY1* and *LEFTY2*	Diaz-Hernandez et al. (2020)
	IVDs dissected from healthy adult human donors. NP and AF cells isolated from tissue	scRNA-seq	Displayed high expression of *SFRP1, BIRC5, CYTL1, ESM1* and *CCNB2* genes in AF cells and *COL2A1, DSC3, COL9A3, COL11A1,* and *ANGPTL7* in NP cells. Identified signature transcription factors for AF (*FOXM1*) and NP (*KDM4E*)	Fernandes et al. (2020)
	IVDs dissected from healthy adult human donors. Cells isolated from IVD tissue	Droplet-based scRNA-seq	Identified major cell clusters in human IVDs. Confirmed *PDGFRA* and *PROCR* marked progenitor cells in IVD.	Gan et al. (2021)
	Discs isolated from adult humans. Primary NP and AF cells isolated and immortalized	cDNA Microarray analysis	Identified of membrane-associated genes including AF-specific *COLEC12, LPAR1* and *CHIC1* and NP-specific *CLDN1, TMEFF2, EFNsA1* and *NETO2*	Minogue et al. (2010); van den Akker et al. (2020)
	AF and NP tissue surgically collected from adult IDD cervical discectomy patients	RNAseq	AF showed higher expression of genes including COL1A2, COL6A1, LAMA3, and DLL1. NP showed higher expression of COMP, LUM, COL2A1 and BGN.	Riester et al. (2018)
	Notochordal and sclerotomal cells isolated from human embryonic and fetal spines (7.5–14 WPC)	Microarray analysis followed by PCA	Identification of notochordal markers including CD24, STMN2, RTN1, PRPH, and CXCL12	Rodrigues-Pinto et al. (2018)
	NP collected from adult human patients with IDD or spinal cord injury	RNA-seq followed by stringent algorithmic pipeline to identify lncRNA	Indicated that 1854 lncRNAs were differentially expressed in IDD including upregulation of EPB41L2, SH3BP2 and ATM and downregulation of SIPA1L2, KMT2E and PAAF1	Zhao et al. (2016)
Mouse	Notochords isolated from Shh-cre; ROSA:YFP mice at E12.5 and P0 days	Whole transcriptome RNA-Seq followed by PCA	Differential expression of Shh, TGF-β, and IGF pathways between E12.5 and P0. Lower mRNA abundance of *Shh, Ptch1, Smo* and *Gli1* at P0	Peck et al. (2017)
	Cells isolated from dissected vertebral column tissue of E12.5 embryonic mice	*In vivo* high-throughput ChIP-Seq	*Pax1* and *Pax9* positively regulate key cartilage development genes including S*ox5, 76an, Col2a1* and *Wwp2*	Sivakamasundari et al. (2017)
	Tissue from adult Sox9^cKO^ mice 5 days post-tamoxifen injection	Microarray analysis followed by PCA	*Sox9* deletion resulted in downregulation of ECM-related genes *Col2a1, Col11a1, Col9a2*, and ion channel-encoding genes *Trpm4, Trpm7* and *Piezo2*, as well as upregulation of matrix remodeling and inflammation genes including *Timp1, Adam10,* and *Cxcr8*	Tsingas et al. (2020)
Bovine	Coccygeal discs collected from 6mo to 12mo old calves	scRNA-seq and bulk RNA-seq	Identified 24 AF-specific markers including *TIE1, CTGF, PCOLCE,* and *HSPA5.* Identified 27 NP-specific markers including *CD24, KRT8, CCN2,* and *HIGD2A*	Calió et al. (2021)
	Coccygeal discs collected from adult cows	RNA *in situ* hybridization and z proportion test	Identified 10 novel markers including *Lam1* and *Thy1* in the AF and *Gli1, Gli3, Noto, Scx, Ptprc, Sox2, Zscan10* and *LOC101904175* in the NP	Li et al. (2019)
	Caudal discs collected from 2 to 3yr old cows	scRNA-seq	Identified AF markers including *MGP, COMP, SPP1*, and *GSN*. Identified NP markers including *CP, S100B, SNORC* and *CRELD2*	Panebianco et al. (2021)
Rat	Cervical IVDs from healthy, adult, wild-type rats. AF and NP cells isolated from tissue	scRNA-seq	Identified highly specific marker genes including Bpifa2f, Mmmp3, and IL11 for IAF; Fibin, Myoc, and Igfbp5 for OAF; Krt7, Prrg4, and Akap12 for NP.	Wang et al. (2021a)
B. Proteomics				
Human	IVDs collected from healthy adult patients	LC-MS/MS and iTRAQ analysis	High levels of lubricin protein and lower levels of biglycan in IVDs compared to other cartilage subtypes	Önnerfjord et al. (2012)
	IVDs collected from human fetal spines (24 WPC)	LC-MS/MS	Identified 1316 proteins and 10 significant protein clusters	Rajasekaran et al. (2020)
	IVDs collected from human fetal (less than 24 WPC) and adult spines	ESI-LC-MS/MS	Identified 14 proteins uniquely expressed or upregulated in fetal IVDs compared to adults. Proteins include P4HA1, P4HA2, Procollagen-lysine, PLOD1, SERPINH1, CD109, LGALS3, SERPINF1, and Annexin A1, A4 and A5	Rajasekaran et al. (2021)
	IVDs collected from adult IDD patients. AF and NP tissues isolated from discs	FTMS/ITMSMS and iTRAQ analysis	In degenerated tissues, identified differential regulation on 73 proteins in the AF and 54 proteins in the NP.	Sarath Babu et al. (2016)
	AF cells collected from adult IDD and scoliosis patients	Silver stained 2-DE gels and MS	In degenerated AF cells, identified decrease in HSPA8Q, G6PD, and protocadherin-23 and increase in GNAI2, superoxide dismutase, TMEM51, adenosine receptor A3, 26S protease regulatory subunit 8, LPPR2, and FAR1	Ye et al. (2015)
	IVDs collected from IDD, AIS and trauma patients (11–53years). AF, NP, and EP isolated from discs	LC-MS/MS and iTRAQ analysis	In degenerated tissues, revealed increased levels of HTRA1, COMP, and CILP in AF and CILP and CILP2 in NP tissue	Yee et al. (2016)
Mouse	IVDs collected from healthy adult *Foxa2* ^*mNE*^ *-Cre/ZEG* mice. NP and AF isolated from tissue	LC-MS/MS	NP cells displayed enrichment in proteins including Cdh2, Dsp, Gja1, Wnk1, Vamp3. AF cells displayed enrichment in proteins including Sod3 and Clu	Kudelko et al. (2021)
	Intact IVDs collected from healthy adult mice	LC-ESI-MS/MS	Identified 1360 proteins in distinct categories. Confirmed presence of 14 previously identified IVD-associated markers	McCann et al. (2015)
Bovine	Caudal discs collected from healthy fetal, 12mo old and 16–18years old cows	LC-MS/MS and iTRAQ analysis	Observed enrichment of Collagen XII and Collagen XIV in fetal NPs, Collagen XI in young NPs and Fibronectin and Prolargin in older NPs	Caldeira et al. (2017)
	Caudal discs collected from 18mos to 24mo old cows	Differential in-gel electrophoresis proteomics (DIGE)	Identified 14 proteins specific to the disc or cartilage cells	Gilson et al. (2010)
C. Metabolomics				
Human	IVDs collected from adult IDD patients. Tissue isolated from discs	^1^H HR MAS NMR spectroscopy	Degraded discs had significantly higher concentrations of multiple metabolites including creatine, glycine, and leucine	Radek et al. (2016)

PCA, principal component analysis; WPC, weeks post-conception; ECM, extracellular matrix; cKO, conditional knock-out; NP, nucleus pulposus; AF, annulus fibrosus; IAF, inner annulus fibrosus; OAF, outer annulus fibrosus; LC, liquid chromatography; MS, mass spectrometry; ESI-MS, electrospray ionization tandem mass spectrometry; 2-DE, 2-D electrophoresis; FTMS, Fourier transform mass spectrometry; ITMS, ion trap tandem mass spectrometry; HR MAS, high-resolution magic angle spinning; NMR, nuclear magnetic resonance.

Expression of PAX1 and PAX9 protein is necessary for spinal column patterning and IVD differentiation (Neubüser et al., 1995). Transcriptomics analysis of transgenic embryonic mouse cells showed that *Pax1* and *Pax9* positively regulate many genes associated with cartilage formation and activate expression of chondrogenic genes during early IVD differentiation (Sivakamasundari et al., 2017). The transcription factors, *Sox5*, *Sox6*, and *Sox9*, known as the *Sox* trio, all contribute to embryonic skeletogenesis and control chondrocyte differentiation and maturation (Lefebvre, 2019). In particular, *Sox9* has been shown to play a major role in the commitment of progenitor cells to the chondrogenic fate. Heterozygous mutations in and around *Sox9* can lead to campomelic dysplasia, a severe form of human chondrodysplasia that affects development of the skeleton, reproductive system, and face (Foster et al., 1994; Wagner et al., 1994). *Sox9* has also been shown to play a specific role in inner AF differentiation during disc development (Bi et al., 1999). Transcriptomics analysis of *Sox9* mutant mice showed that deletion of *Sox9* resulted in progressive disc degeneration and alterations in ECM organization indicating that *Sox9* expression can also directly impact disc cell survival and ECM maintenance (Tsingas et al., 2020). Mammalian homeobox gene *Bapx1* (Nkx3.2) was shown to suppress hypertrophic differentiation of chondrocytes in conjunction with *Sox9* since they regulate similar pathways in the same tissues (Chatterjee et al., 2014). Ultimately, Chatterjee et al. found that *Bapx1* and *Sox9* co-regulate 137 genes that are involved in chondrocyte differentiation.

*Shh* expression, previously identified to be critically important for embryonic notochordal cell differentiation, was found to be significantly downregulated in early postnatal NP cells (Peck et al., 2017). Using RNA-Seq with the *Shh*-Cre mouse model, Peck et al. found patterning signals like *Shh* expression were downregulated over time while expression of synthetic genes like TGF-β and insulin growth factor (IGF) were upregulated in the same developmental window, providing insights into gene families required for patterning, ECM synthesis, and maintenance of the NP.

In addition to extensive transcriptomics studies in mouse models, RNA-Seq studies of isolated human embryonic and fetal notochordal cells have identified temporally specific notochord markers such as *CD24*, stathmin-2 (*STMN2*), and reticulon 1 (*RTN1*), and implicated genes involved in inhibition of vascularization and inflammation as important master regulators of notochordal cell gene expression (Rodrigues-Pinto et al., 2018). Signaling network analysis on healthy human IVD cells also found that platelet-derived growth factor (PDGF) and TGF-β cascades are necessary cues in the formation and maintenance of the NP microenvironment (Gan et al., 2021). A single-cell RNA-Seq (scRNA-Seq) study analyzed primary NP and AF cells isolated from healthy adult discs and observed that receptor signaling pathway genes were highly expressed in the AF, whereas protein synthesis and ECM genes were highly expressed in the NP (Fernandes et al., 2020). In this study, Forkhead box M1 (*FOXM1*) and lysine demethylase 4E expression (*KDM4E*) were identified as signature gene markers for the AF and NP respectively. Furthermore, a membranome-based approach on immortalized NP and AF cell lines established from adolescent healthy disc tissue yielded various membrane-associated markers in the AF and NP (van den Akker et al., 2020).

While bulk RNA-Seq has provided key insights into global gene expression profile changes that occur during the development of the IVD, it is important to account for the significant cellular heterogeneity that is present in fully formed IVD. Thus, single cell analyses such as single cell RNA-Seq (scRNA-Seq) can deconvolute some of these differences that may be lost when averaging the entire cellular population, especially at later time points (Kraus et al., 2019). Kraus et al. highlighted the importance of single-cell sequencing through the plate *in situ* hybridization (PISH) assay, where the cells were first quantified using a population average then assessed using single-cell analysis. They were able to detect differences between the NP, AF, and transition zone in between the NP and AF. Furthermore, the center region of the NP exhibits additional heterogeneity as indicated by the presence of a distinctive set of transcripts that are involved in cell pluripotency (*Sox2* and *Oct4*) and the development of the notochord (*Noto* and *T*) and axial skeleton (*Shh* and *Gli3*).

Transcriptomics has also been used to identify biomarkers and potential therapeutic targets for IDD. One study analyzed the rat IVD transcriptome using scRNA-Seq and established a comprehensive single-cell gene expression map as well as potential new genetic markers for IVD and multi-functional stem cells (Wang J. et al., 2021). Due to their similarity in size and morphology to human IVD, bovine discs have been popular experimental models in IVD research. RNA *in situ* hybridization of adult bovine coccygeal IVDs identified 10 novel biomarkers, and further scRNA analysis of bovine calf coccygeal IVDs revealed 27 NP specific genes, 24 AF specific genes, and various cell population clusters (Li et al., 2019; Calió et al., 2021) (biomarkers and genes listed in Table 3). scRNA analysis of bovine calf caudal IVDs identified multiple biomarkers and 15 unique cell clusters of potential IVD progenitor cells (Panebianco et al., 2021). Analysis of notochordal cells derived from human embryonic stem cells identified a regulatory network for notochordal differentiation (Diaz-Hernandez et al., 2020). RNA-Seq of NP samples from human adult patients with IDD was used for genome-wide identification of long noncoding RNAs (lncRNAs) and competing endogenous RNAs (ceRNA) that may serve as diagnostic and therapeutic biomarkers (Zhao et al., 2016). Degenerative disc sequencing on adult human tissue also identified ECM regulatory networks and observed that ECM related genes were associated with multiple growth factors (Riester et al., 2018).

Proteomics is used to identify and quantify proteins in a cell, tissue, or organism and to understand the structure and functions of these proteins (Aslam et al., 2017). Using proteomics technologies, researchers have made great inroads into understanding and optimizing animal models of IVD development, maintenance, and degeneration. Comprehensive proteomic profiles of the healthy mouse IVD have been established using mass spectrometry methods to identify differences in protein expression between the NP and the AF (McCann et al., 2015; Kudelko et al., 2021). Difference gel electrophoresis (DIGE) analysis of the NP from young bovine caudal discs also suggested that subpopulations of notochordal-like cells remain in the mature bovine discs and that these cells may play a role in disc repair and survival (Gilson et al., 2010). These datasets have also been compared to protein expression profiles from human disc samples to assess the relevance of mouse and bovine models for IVD research. Liquid chromatography tandem mass spectrometry (LC-MS/MS) conducted on human fetal IVD samples also identified 10 protein clusters and proteome networks involved in IVD growth and development (Rajasekaran et al., 2020). Proteomics analyses have also established differential expression of proteins between normal and degenerative discs to identify potential therapeutic targets. One study utilized electrophoresis, mass spectrometric analysis, and database research to compare the protein expression profiles of normal and degenerated human AF and found 10 proteins that had highly altered expression between the two groups (Ye et al., 2015). A later study using isobaric tag for relative and absolute quantitation (iTRAQ) and one-dimensional gel electrophoresis-Fourier transform mass spectrometry/ion trap tandem mass spectrometry (FTMS/ITMSMS) that compared normal and degenerative discs showed that in patient samples, 73 proteins in the AF and 54 proteins in the NP exhibited differential expression levels between the two groups (Sarath Babu et al., 2016).

Notably, Tam et al. were able to establish a spatiotemporal proteomics atlas of human intervertebral discs, DIPPER, through various measurement types including label-free proteomics, transcriptomics, and SILAC to degradome (Tam et al., 2020). Two cadaver lumbar samples from a young and an aged cadaver were used to establish a high-resolution point-reference map of static spatial proteomes along the lateral and anteroposterior directions of IVDs at three lumbar levels. They also determined that the point-reference genome-wide profile has clinical relevance since it integrates with the dynamic proteome and transcriptome of clinical samples. They showed that the IVD is comprised of 3,100 proteins, encompassing roughly 400 matrisome proteins and 2,700 non-matrisome proteins, with 1,769 proteins detected in three or more profiles. When this dataset was analyzed, they were able to conclude that the different regions of the IVD (NP, NP/inner AF transition zone, inner AF, AF, and outer AF) had unique protein profiles in healthy IVDs. These profiles will change as the IVD ages, most notably the inner AF becoming gradually similar to the outer AF. Kudelko et al. also performed extensive and comprehensive proteomic profiling in murine models that mimicked a healthy young adult IVD to generate PRIMUS (Proteomic Resource for Intervertebral disc of MUS musculus) (Kudelko et al., 2021). Their findings were consistent to that of the DIPPER data set, where they noted that both the AF and NP have unique profiles. Structurally, the AF in mice and humans are similar since the organized lamellar structure is preserved. In contrast, the NP in mice have a highly cellularity, while the human NP have a greater proportion of ECM.

Both transcriptomic and proteomic profiles of the developing disc have established the unique origin of the NP in mouse and human samples. However, the substantial structural changes that occur in the development of the IVD and the inherent heterogeneity of the fully formed NP, including the differences in cell population between mouse and human samples have made distinctly defining the ultimate mature NP cell profile a difficult task. In a recent study in adult human NP cells, Richardson et al. focused specifically on measuring putative notochordal and NP cell markers and found expression levels of previously identified novel NP and notochordal markers such as *KRT8*, *KRT18*, *KRT19*, *CA12*, *FOXA2*, and *NOG* stayed consistent regardless of donor age or degeneration (Richardson et al., 2017). Additional investigations verifying the persistence of expression of previously identified markers of NP cells throughout development and ageing and large-scale comparative studies between animal and human notochordal/NP cell omics datasets will lead to the establishment of a consensus list of mature NP cell markers in the future.

In addition to well-established transcriptomic and proteomic studies, other types of large-scale omics investigations are gaining momentum in IVD research as technologies become further refined, particularly in important mouse model studies (Veras et al., 2020). Matrisome and metabolome-centered inquiries are examples of such analyses. The matrisome is a collection of genes encoding for the ECM and ECM-associated proteins and allows for better understanding of both development and degeneration (Hynes and Naba, 2012; Naba et al., 2016). One study utilized iTRAQ LC-MS/MS analysis on fetal, young, and old bovine discs to define the NP matrisome (Caldeira et al., 2017). The study found an enrichment of collagen types XII and XIV in fetal discs and fibronectin and prolargin in aged discs and provided a useful database for reference in understanding development and identifying therapeutic targets in the context of the IVD ECM. Matrisome analysis on degenerated adult human samples showed fibrosis-like changes in the ECM due to ageing and degeneration as well as decreased solubility and changes in fibril diameter (Yee et al., 2016). Degeneration also resulted in the upregulation of collagen biosynthesis pathways and genes associated with tissue development and cell death regulation (Rajasekaran et al., 2021).

Metabolomics is the utilization of advanced analysis techniques to identify and characterize metabolites from cells and tissues (Wishart, 2019). Metabolomics have been conducted on human degenerative tissues to identify the changes that occur in pathologic conditions to biological metabolic mechanisms (Swank et al., 2020). One study conducted high-resolution magic angle spinning nuclear magnetic resonance (HR-MAS NMR) spectroscopy on degenerated adult human lumbar discs and demonstrated a correlation between changing metabolite concentrations and degrees of degeneration (Radek et al., 2016). Identifying pathologic alterations to metabolites will be applicable to the development of novel diagnostics and therapeutics. As a corollary to omics studies, large clinical data analyses and artificial intelligence (AI) networks for analyzing large scale, global patient population data on low back pain are important disciplines that have garnered much interest in recent years and continue to advance with the advent of more sophisticated analysis methods. Studies have shown that lower back pain is highly prevalent among the elderly population and causes more global disability than any other condition (Hoy et al., 2014; de Souza et al., 2019). Population studies have also been used to develop prediction models and risk scores for lower back pain (Mukasa and Sung, 2020).

## Discussion

The IVD is an indispensable structure in the spine. The three substructures within the IVD work in concert to provide both mechanical support and biological function in the spinal column. The aggregated efforts of many labs over several years have established a solid framework for our understanding of the IVD. As a field, we have made great strides into elucidating many mechanisms that drive IVD development as well as mechanical and biological stresses that lead to IDD and related pathogenesis. While many early genetic and lineage tracing investigations laid the critical foundation for this field, advancements in omics techniques in more recent years have provided global overviews into early embryonic development, the genetic and molecular profile of a healthy IVD vs. a degenerated IVD, and importantly, new strategies in diagnostics and potential disc regeneration therapies.

The complexity of the IVD, in development, composition, and function, makes this unique structure a particularly difficult one to fully understand. As the majority of the human population globally will be afflicted by disc degeneration and associated pain and other complications in their lifetimes, it is critically important to establish thorough and comprehensive molecular profiles of the IVD at various stages of development and disease. With the application of advancing omics technologies to both animal models and human samples, we already have an unprecedented and detailed view of many aspects of the IVD. While animal models are by no means a perfect replication of the human disc due to differences in IVD size, cell turnover, and biomechanical load, they have served as important starting points for developmental and degenerative studies as well as preclinical studies for therapeutics. Although human patient studies are difficult to achieve in large sample sizes, the ability to compare human data to large sets of animal data allows investigators to more readily establish validity of and relevance within their findings.

The ideal outcome for future regenerative therapies is to be able to fully replace any damaged and diseased tissue with a substitute that has the capability to restore physiological function throughout the remainder of the patient’s lifetime. The best way to achieve this goal is to recapitulate the composition of the native tissue as closely as possible. Considering that organs and tissues in the body are in their healthiest state at the earliest points following completion of development and that the ultimate objective is to replicate native functional tissue, a key ingredient that is necessary to make significant progress towards building successful IVD regenerative strategies is a fully elucidated genetic and molecular profile of IVD development. Although multiple approaches to regenerative therapeutics have been under investigation for several years, we are still missing a comprehensive methodology that combines cellular gene expression, molecular signaling, and the ECM structure to construct native tissue-like replacements. Building on genetic and omics studies that have been completed thus far, in combination with multi-omics approaches, which allow for both temporal and spatial deconvolution of gene, protein, and other biomolecule expression at unprecedented resolution, we are well on our way to building a comprehensive profile of many aspects of the IVD. Taken together, these data will be crucial in furthering our understanding of development, maintenance, and pathology of the IVD, and will inform novel diagnostic and therapeutic advances in the future.
