# Supplementary material for: Development, Pathogenesis, and Regeneration of the Intervertebral Disc: Current and Future Insights Spanning Traditional to Omics Methods
Source: Front Cell Dev Biol. 2022 Mar 11;10:841831. doi: 10.3389/fcell.2022.841831 (PMC8963184; doi:10.3389/fcell.2022.841831)
Supplement: Supplementary file 1 [file Table1.docx]

**Supplementary Table 1**

| **Material** | **Type** | **Specific Study** | **Tissue/ Model** | **Result** | **Reference** |
| --- | --- | --- | --- | --- | --- |
| Natural Hydrogel | Alginate Hydrogel | Bone marrow aspirate concentrated containing ultra-purified alginate hydrogel | Rabbit IVD degeneration model | Gel significantly enhanced repair of IVD defects | (Ukeba et al., 2021) |
|  |  | Alginate hydrogel cocultured with chitosan hydrogel | NP and bone marrow stem cells isolated from porcine IVDs | Alginate more readily supports cell viability and matrix accumulation | (Naqvi and Buckley, 2015) |
|  | Chitosan Hydrogel | Cellulose nanofiber filled chitosan hydrogel | Implemented in AF of Porcine IVD degeneration model | Restored IVD biomechanics and support cell growth | (Doench et al., 2019) |
|  |  | Injectable chitosan carboxymethylcellulose hydrogel system with encapsulated ASCs | Long-term (12 mos) ovine IVD degeneration model | Stabilized disc height and significantly decreased degeneration progression | (Schmitt et al., 2021) |
|  | Chondroitin Sulfate (CS) Hydrogel | CS incorporation into disc-derived self-assembled ECM hydrogel | Nasal chondrocytes isolated from porcine nasal tissue | CS incorporation increased sulfated GAG production and Type II Collagen synthesis | (Borrelli and Buckley, 2020) |
|  |  | Cross-linked CS Hydrogel | Rabbit IVD degeneration model | Intradiscal administration appears to inhibit degenerative cascade of IDD | (Nakashima et al., 2009) |
|  | Fibrinogen | Tetrafunctional ethylene oxide/propylene oxide block copolymer (Tetronic1307) | MSCs from porcine lumbar IVDs | Elastic matrix modulus influences differentiation of NP-SCs to chondrogenic (low modulus) or osteogenic (high modulus) fate | (Navaro et al., 2015) |
|  | Hyaluronate Hydrogel | Cross-linked hyaluronate hydrogel | Rabbit IVD degeneration model | Intradiscal administration appears to inhibit degenerative cascade of IDD | (Nakashima et al., 2009) |
|  |  | Platelet-rich plasma and hyaluronic acid hydrogel carrier for human MSCs | Bovine tail IVDs | The hydrogel is a good carrier for MSCs and maintains good cell viability while stimulating cell activity and NP marker expression | (Russo et al., 2021) |
| Synthetic Hydrogel | Polyethylene Glycol (PEG) | PEG dimethacrylate nano-fibrillated cellulose | Bovine tail IVDs | Hydrogel was able to mimic mechanical and functional properties of NP | (Schmocker et al., 2016) |
|  |  | Maleimide terminated 8-arm star PEG with IKVAV and AG73 peptides | Human NP cells | Controlling peptide selection and presentation can lead to a more juvenile NP cell phenotype that is not dependent on substrate stiffness | (Barcellona et al., 2020) |
|  |  | Laminin-111 functionalized PEG Hydrogel (PEG-LM111) | NP cells isolated from porcine lumbar IVDs | Gel carrier significantly improved NP cell retention in disc space | (Francisco et al., 2013) |
|  |  | Simvastatin-loaded PEG-PLGA-PEG gel | Rat tail disc needle injury model | Injection increased aggrecan expression and sulfated GAG content as well as significantly reversing damage and improving histological change from needle puncture | (Zhang et al., 2009) |
|  | Polyurethane (PU) | Injectable silk fibroin/polyurethane composite hydrogel (SF/PU) | Porcine lumbar IVDs | Hydrogel increased cell viability and proliferation and can be used as a NP replacement in IDD treatment | (Hu et al., 2012) |
| Interpenetrating networks (IPN) | IPN Hydrogel | IPN hydrogel comprised of dextran, gelatin, and PEG | NPCs from rat lumbar and tail IVDs and porcine lumbar IVDs for use in  degeneration model | IPN can provide template for rehydration regeneration for degenerative NPs and supports long-term cell retention and survival | (Gan et al., 2017) |
|  |  | Non-covalent hydrogel with crosslinked HA-BDDE and HA-pNIPAM | NP cells isolated from bovine tail IVDs | Gel supported cell viability and IVD matrix production | (Guo et al., 2022) |
|  |  | IPN hydrogel comprised of dextran, chitosan, and teleostean (DCT) | Moderately degenerated goat lumbar IVDs | Restores mechanical function in ex vivo IDD model, supports matrix producing capacity of NP cells and MSCs, and shows safe retention in in vivo model | (Gullbrand et al., 2017b) |
|  | Semi-IPN | Collagen-low molecular weight hyaluronic acid semi-IPN loaded with gelatin MS | MSCs isolated from human bone marrow | Supported growth and chondrogenic differentiation of MSCs and nasal chondrocytes | (Tsaryk et al., 2015) |
| Nanofibers/ Scaffolds | Nanofiber scaffold | Link N Nanofiber Scaffolds (LN-NS) | Rabbit NP cells | Had excellent biocompatibility and bioactivity with NPCs | (Wang et al., 2012) |
|  |  | Pro-Hyp-Gly collagen peptide presenting nanofiber scaffold | Rabbit IVD degeneration model | Scaffold induced more GAG and collagen deposition as well as providing functional recovery of the IVD degeneration. | (Uysal et al., 2019) |
|  | Polyurethane (PU) | PU Scaffolds | Bovine whole IVD organ culture model using bovine caudal NPCs and human MSCs | Delay further degradation of pre-existing disc tissue and restore the mechanical properties of nucleotomized  IVDs | (Li et al., 2016) |
|  |  | PU Scaffolds | IVD tissue isolated from human donors | Scaffolds helped synthesis of appropriate ECM and mRNA expression of chondrogenic and NP specific markers | (Mauth et al., 2009) |
|  | Poly (Ether carbonate urethane) Urea (PECUU) | Electrospun PECUU Fibrous scaffolds | AF stem cells obtained from rabbit IVDs | AF cells cultured on scaffolds differentiated into AF-like cells | (Zhu et al., 2016) |
|  |  | Decellularized AF matrix/PECUU electrospun scaffolds | AF derived stem cells | AF cells proliferated well on scaffolds and had increased collagen and aggrecan expression | (Liu et al., 2020) |
|  | PTMC scaffold | PTMC Scaffold seeded with BM-MSCs and covered with Poly(ester-urethane) membrane | Bovine organ culture annulotomy model | Has potential to hinder NP herniation, stabilize disc height and positively modulate cell phenotype of native disc tissue | (Pirvu et al., 2015) |
| Microspheres | Spongy microspheres | Nanofibrous spongy microspheres (NF-SMS) | Rabbit MSCs used to study degeneration in lumbar region | Increase MSC adhesion, proliferation, and differentiation to NP-phenotype | (Feng et al., 2020) |
|  |  | Nanofibrous spongy microspheres (NF-SMS) and pDNA-carrying polyplex encapsulating biodegradable nanospheres | Intradiscal injection in rat tail | pDNA reduced pathogenic fibrosis of NP tissue in degeneration model and delivery system supports IVD regeneration | (Feng et al., 2017b) |
|  | PLGA constructs | 3D PLGA constructs loaded with dexamethasone and growth factor embedded heparin/poly(L-lysine) nanoparticles | Rat MSCs isolated from bone marrow | Promote proliferation of MSCs and improves differentiation into NP-like cells | (Liang et al., 2012) |
|  | Hydrogel microspheres | Platelet-rich plasma implanted gelatin hydrogel microspheres | Rabbit IVD degeneration model | Suppressed IVD degeneration progress and showed increase of proteoglycan in NP and AF | (Nagae et al., 2007) |
|  | Hydrogel microspheres | GDF5-loaded gelatin methacryloyl (GelMA) microspheres (GM) | Rat adipose-derived MSCs | Delivery method enhanced differentiation into NP-like cells, maintained NP tissue integrity and accelerated ECM synthesis | (Xu et al., 2020b) |
| Natural Scaffolds | Silk Scaffolds | Silk fibroin scaffolds. Some enhanced with RGD-peptides | AF cells dissected from 6-9 mo bovine caudal IVDs | Appropriate scaffold to grow AF cells. Cells had higher collagen and aggrecan levels. RGD addition influenced cell morphology | (Chang et al., 2007) |
|  |  | Silk fibroin biphasic scaffold | AF and NP cells isolated from rabbits | Appropriate scaffold for growth of AF and NP cells | (Du et al., 2014) |
|  |  | Porous silk fibroin scaffolds | NP cells isolated from rabbits | Scaffolds provided an appropriate environment for NP cell adhesion, proliferation, and infiltration | (Zeng et al., 2014) |

**Supplemental Table 1:** **Summary of regenerative materials for IVD therapeutics.** MSC: Mesenchymal Stromal Cell; ASC: Adipose Derived Stem Cells; NP: Nucleus Pulposus; AF: Annulus fibrosus; BM: Bone Marrow; ECM: Extracellular matrix; PU: Polyurethane; PEG: Polyethylene Glycol; PLGA: Poly(lactide-co-glycolide); PTMC: Poly(trimethylene carbonate); MS: Microspheres; GAG: Glycosaminoglycans
